# Fidelity and feasibility of a pilot implementation of adapted WHO digital interventions to scale up mental health in Nigerian primary care: A mixed-methods process evaluation

**DOI:** 10.1017/gmh.2026.10131

**Published:** 2026-01-22

**Authors:** Abiodun O. Adewuya, Jibril Abdulmalik, Seye Abimbola, Olabisi Oladipo, Falmata Shettima, Asmau Dahiru, Adedolapo Fasawe

**Affiliations:** 1 Behavioural Medicine, Lagos State University College of Medicine, Nigeria; 2 University of Ibadan College of Medicine, Nigeria; 3 The University of Sydney School of Public Health, Australia; 4 Center for Mental Health Research and Initiative, Nigeria; 5 Federal Neuropsychiatric Hospital Maiduguri, Nigeria; 6 Lagos State Government Ministry of Health, Nigeria

**Keywords:** implementation science, digital health, global mental health, primary care, Nigeria

## Abstract

To address challenges in the real-world implementation of digital health for mental healthcare in Nigeria, this study conducted a process evaluation of five World Health Organization-recommended digital tools within a state-wide primary health care program in Lagos. Employing a convergent mixed-methods design across five facilities, we measured implementation fidelity through observation and platform analytics, and assessed stakeholder perceptions via validated surveys and interviews. The findings revealed a sharp divergence in success. Administrative tools that streamlined workflows, such as drug stock notification and automated client reminders, achieved high fidelity (>90% adherence). In contrast, clinical tools that altered provider–patient interactions, including a decision support app and a client helpline, demonstrated low fidelity (<66% adherence). Qualitative analysis attributed this gap to the successful tools’ seamless workflow integration versus the clinical tools’ disruption of practice and introduction of perceived professional and liability risks. The study concludes that digital health adoption is determined less by technological sophistication than by its integration into human systems. Scaling these innovations effectively requires prioritizing tools that align with existing workflows and developing a supportive policy ecosystem to address the professional concerns of frontline health workers.

## Impact statement

In many parts of the world, including Nigeria, there is a major gap in mental healthcare. Digital tools, like smartphone apps and text messages, offer a promising way to help primary care workers deliver this much-needed support. However, simply introducing new technology does not guarantee success. Our research, conducted in primary care clinics in Lagos, aimed to understand why some digital health tools are adopted while others are left behind.

We discovered a critical difference: tools that simplified administrative tasks, such as tracking medicine inventory or sending automatic appointment reminders, were highly successful and embraced by health workers. In contrast, tools that altered the clinical interaction with patients, such as a digital guide used during consultations or a 24/7 helpline, faced significant resistance. The reason was not the technology itself, but its impact on people. Health workers felt these clinical tools disrupted their relationship with patients and introduced new professional anxieties and legal risks without clear support or guidelines.

This study provides a vital lesson for global health: for digital interventions to succeed, they must be designed with and for the frontline workers who use them. Our findings offer a practical roadmap for policymakers and health program managers, emphasizing that success hinges on integrating technology seamlessly into existing workflows and creating a supportive policy environment that addresses the real-world concerns of healthcare providers. This human-centered approach is essential for harnessing the true potential of digital health to close the mental health gap in Nigeria and in similar settings worldwide.

## Introduction

The burden of mental, neurological and substance use disorders represents a substantial global health challenge, contributing to ~14% of the worldwide burden of disease (Vos et al., 2020). This epidemiological reality is particularly pronounced in low- and middle-income countries (LMICs), where a profound and persistent mental health treatment gap leaves the vast majority of affected individuals without access to even basic care (Patel et al., 2018). In sub-Saharan Africa, this gap is especially stark. Nigeria, the continent’s most populous nation, exemplifies these challenges; a significant shortage of mental healthcare professionals poses a major obstacle to service scale-up, and studies indicate that up to 85% of individuals experiencing significant mental health problems receive no formal treatment, leading to severe disability and substantial social and economic costs (Saraceno et al., 2007; Charlson et al., 2014). Within the megacity of Lagos, an urban metropolis with a population exceeding 20 million, the Lagos State Mental Health Survey revealed high prevalence rates of common mental disorders and elevated suicide risk among residents, highlighting the urgent need for systematic service delivery improvements (Adewuya et al., 2016, 2018).

In response to these challenges, a key global strategy has been the integration of mental health services into primary care systems. The World Health Organization’s (WHO) Mental Health Gap Action Programme (mhGAP) was introduced to facilitate this integration, providing evidence-based guidelines for nonspecialist health workers (WHO, 2008). Lagos State subsequently adopted this framework through the Mental Health in Primary Care (MeHPriC) program, which utilizes a task-shifting approach to enable nurses and community health officers to deliver evidence-based psychosocial interventions (Adewuya et al., 2019a, 2019b, 2025). While initial evaluations of MeHPriC demonstrated both clinical and cost-effectiveness, implementation reviews have identified persistent challenges that threaten the program’s sustainability and scale-up. These include geographical inaccessibility for remote clients, insufficient demand generation, delayed care provision and suboptimal adherence to clinical protocols; factors that collectively limit the program’s capacity to address coverage and quality gaps (Adewuya et al., 2025).

Digital health technologies, particularly mobile health (mHealth), have been proposed as a veritable platform to bridge these treatment gaps (Onu and Onyeka, 2024). Such technologies could leverage Nigeria’s robust mobile infrastructure, with over 188 million active lines and 48% broadband penetration, to enhance service delivery through tools like automated reminders and decision support (Labrique et al., 2013; Nigerian Communications Commission, 2021). Research in Nigeria suggests that mobile phone interventions can improve treatment engagement and adherence. Studies among perinatal adolescents have demonstrated that mobile phone-delivered interventions significantly increase attendance at antenatal clinic appointments and improve adherence to mental health treatment information (Kola, 2019; Kola et al., 2021), while similar interventions among older adults with mental illness reduced dropout from outpatient services (Elugbadebo et al., 2023) and improved outcomes for adolescents with perinatal depression (Gureje et al., 2022). The WHO’s “Guideline Recommendations on Digital Interventions for Health System Strengthening” (WHO-DIHS) provides evidence-based recommendations for such interventions, including drug stock notifications and electronic clinical algorithms, which could mitigate MeHPriC’s logistical and clinical constraints (WHO, 2019). Indeed, prior research within the MeHPriC project demonstrated that adding mobile telephone support significantly improved adherence and clinical outcomes, and was both cost-effective and acceptable to clients (Adewuya et al., 2019b). Recent studies in Nigeria have shown that app-based format of the mhGAP-IG has the potential to substantially aid the scale-up of mental health services (Kohrt et al., 2025).

However, the transformative promise of digital health is tempered by critical perspectives highlighting significant implementation barriers. Infrastructural inequities, such as unreliable electricity and inconsistent network connectivity, may exacerbate access disparities (Kruk et al., 2018). Qualitative studies in Nigeria have identified further disadvantages, including stakeholder skepticism regarding digital tool efficacy and sustainability (Chen and Gombay, 2024), systemic challenges in integrating digital health technologies (Egwudo et al., 2025), barriers to telemedicine provision encompassing technological and regulatory constraints (Cole et al., 2025) and limited accessibility to technology and internet connectivity for some populations (Bawa-Muhammad, 2025). Moreover, disparities in perceptions among key stakeholders, such as policymakers, healthcare professionals and system developers, can foster resistance to new mHealth solutions (Fox et al., 2020). Real-world analogs, such as pilots of the mhGAP application in Nigeria, have reported only moderate implementation success, revealing challenges like low digital literacy among health workers and significant workflow disruptions that necessitate context-specific adaptations (Taylor Salisbury et al., 2021; Ojagbemi et al., 2022).

When a promising, evidence-based intervention like MeHPriC fails to achieve its full potential, or when a new digital tool fails to produce expected benefits, a critical ambiguity arises that poses a central problem for implementation science: was the failure due to an inherently flawed or ineffective intervention (theory failure), or did a potentially effective intervention fail because it was poorly or inconsistently delivered (implementation failure)? (Damschroder et al., 2009). Without a deep understanding of the implementation process itself, it is impossible to distinguish between these two scenarios, leading to the premature abandonment of valuable innovations or the wasteful scaling of interventions that are implemented in name only.

To address this challenge, a rigorous process evaluation focused on implementation fidelity is required. Implementation fidelity, defined as the degree to which an intervention is delivered as it was designed, is a critical determinant of outcomes (Carroll et al., 2007). However, fidelity alone does not capture the full picture. Interventions may be delivered as designed but fail to achieve uptake if they are perceived as unacceptable, inappropriate or infeasible by end-users (Proctor et al., 2011). These implementation outcomes provide a crucial lens into the stakeholder experiences that shape long-term sustainability.

This study, therefore, aims to provide a comprehensive process evaluation of the pilot implementation of five adapted digital health recommendations from the WHO-DIHS (WHO, 2019). Guided by an integrated theoretical framework, the specific objectives of this paper are to: (1) systematically measure the implementation fidelity of the five digital health recommendations; (2) assess their acceptability, appropriateness and feasibility among health workers, clients and health system managers; and (3) use a mixed-methods approach to identify the contextual barriers and facilitators that explain the observed implementation outcomes, thereby providing actionable insights for scaling digital health innovations in similar LMIC contexts. This study was embedded within the broader Research to Enhance the Adaptation and Implementation of Health Systems Guidelines initiative by the Alliance for Health Policy and Systems Research, aiming to strengthen digital health systems in LMICs, with a focus on aligning global guidelines with local system realities.

## Methods

### Study design and theoretical framework

This process evaluation employed a convergent mixed-methods design, nested within a larger implementation study guided by the Knowledge to Action framework (Graham et al., 2006). This approach was selected to facilitate a comprehensive and triangulated understanding of the implementation dynamics by concurrently collecting, analyzing and integrating quantitative and qualitative data (Creswell and Plano Clark, 2018). The study was specifically designed to assess implementation fidelity as a primary outcome, supplemented by a thorough evaluation of acceptability, appropriateness and feasibility among multiple stakeholder groups. The evaluation occurred over a 3-month pilot implementation period, a timeframe considered sufficient for assessing initial adoption patterns and early sustainability indicators in pilot studies (Leon et al., 2011).

The theoretical foundation of this study integrated two complementary implementation science frameworks to provide a holistic view of both the implementation process and its reception by stakeholders. First, we utilized Carroll et al.’s (2007) conceptual framework for implementation fidelity. This framework conceptualizes fidelity as a multifaceted construct comprising *adherence* (whether intervention components were delivered as prescribed), *dose* (how much of the intervention was delivered) and the *moderating factors* that influence these components, including intervention complexity, facilitation strategies, quality of delivery and participant responsiveness. Second, this fidelity assessment was integrated with Proctor and colleagues’ (2011) taxonomy of implementation outcomes. This framework provided the structure for systematically measuring stakeholder perceptions of acceptability, appropriateness and feasibility, which are critical proximal indicators of implementation success that influence longer-term outcomes like sustainability. This dual-framework approach allowed for a robust analysis of not only *if* the interventions were delivered as intended, but also *how* and *why* they were received by end-users.

### Setting and context

The study was conducted within the Lagos State public health system in Nigeria, a megacity with a complex health landscape comprising over 200 primary healthcare centers (PHCs). The pilot was situated within five PHC facilities that were active participants in the established MeHPriC program (Adewuya et al., 2019a, 2019b, 2025). This program provided an advantageous context, as participating facilities possessed established mental health service delivery protocols, health workers trained in task-shifting approaches and existing supervision structures.

### Participants and sampling strategy

A multilevel sampling strategy was employed to recruit three key stakeholder groups. Five PHC facilities were selected through stratified random sampling to ensure representation across Lagos State’s five administrative districts. From a list of all MeHPriC-participating facilities within each district, one facility was randomly selected using a computer-generated number.

- *Health worker participants*: All health workers (*N* = 55) involved in mental health service delivery within the five selected facilities were eligible for inclusion. This sample comprised PHC doctors (*n* = 10), nurses and midwives (*n* = 25), community health officers (*n* = 10) and pharmacy technicians (*n* = 10).
- *Client participants*: A total of 150 clients were recruited through systematic sampling from individuals receiving mental health services. The sampling frame consisted of 752 active clients registered across the five PHC facilities (*N* = 752). To achieve the target sample of 150 participants, we calculated a sampling interval (*k*) of 5, determined by dividing the population size by the desired sample size (752/150 ≈ 5.01). The sampling frame was organized chronologically according to each client’s initial registration date at their respective facility, which provided a natural temporal ordering that minimized selection bias. A random start point was selected from the first five clients on each facility’s register, and, subsequently, every fifth client was invited to participate until the facility-specific quota was achieved. This systematic approach ensured proportional representation across facilities while maintaining methodological rigor. Eligible clients were adults aged 18 years or older with the capacity to provide informed consent. Exclusion criteria included acute psychotic episodes or severe cognitive impairment that would preclude comprehension of the study; these conditions were determined through clinical evaluation by the attending physician or senior nurse at the point of recruitment based on the client’s presentation and ability to provide informed consent. This protocol was reviewed and approved by facility clinicians to ensure participant safety and data quality while maintaining ethical standards
- *Health manager participants*: Five health managers were purposively selected using predetermined criteria to ensure representation of critical implementation decision-making levels. The key informant selection criteria required: (1) direct supervisory responsibility for mental health service delivery within the pilot facilities, (2) minimum 1 year experience in current management role, (3) involvement in at least three supervision cycles during the baseline period and (4) decision-making authority over resource allocation or clinical protocols. The sample comprised three facility in-charges (one per peri-urban facility, representing frontline management), one district-level health coordinator (overseeing the two urban facilities) and one state-level mental health program manager (responsible for policy implementation across Lagos State). This five-person sample was deliberately chosen to capture implementation perspectives across three hierarchical tiers while remaining methodologically feasible for intensive qualitative data collection.

### Intervention description

Five recommendations from the WHO DIHS (WHO, 2019) were selected for pilot implementation based on stakeholder prioritization workshops conducted during a preceding adaptation phase. These interventions were chosen to address known gaps in the existing MeHPriC program.

1. *Drug Stock Notification*: A digital system utilizing SMS, WhatsApp and a digital dashboard for pharmacy technicians to track psychotropic medication inventories, monitor supply levels and generate automated alerts for low stock.
2. *Client-to-Provider Communication*: A dedicated 24-h helpline accessible via voice calls, SMS and WhatsApp for clients experiencing psychological emergencies or requiring urgent clinical advice.
3. *Health Workers’ Supervision*: A portion of traditional face-to-face monthly supervisory visits was replaced with digital supervision conducted via WhatsApp video calls or other secure platforms.
4. *Targeted Client Communication*: An automated messaging system that sent personalized appointment reminders, medication adherence prompts and psychoeducational information to clients via SMS and WhatsApp. This type of mobile phone intervention has previously been shown to reduce dropout from mental health services for older adults in Nigeria (Elugbadebo et al., 2023) and improve adherence for depression management within the MeHPriC program (Adewuya et al., 2019a).
5. *Clinical Decision Support*: Health workers were provided with tablets and mobile phones preloaded with an electronic version of the mhGAP Intervention Guide (e-mhGAP-IG) and other local clinical protocols. The selection of this tool was informed by ongoing efforts to assess the feasibility of the e-mhGAP-IG application for improving the quality of care in Nigerian primary health clinics (Taylor Salisbury et al., 2021; Ojagbemi et al., 2022; Kohrt et al., 2025).

### Implementation process

Implementation commenced with a comprehensive 2-week training program for all 55 health workers. The training employed competency-based assessment methods, requiring participants to demonstrate proficiency in using each digital tool before the pilot began (Michie et al., 2013). Each facility received the necessary hardware (tablets and smartphones) and a budget for data connectivity. Throughout the 3-month pilot, a dedicated technical support helpline and weekly on-site visits from a technical coordinator provided ongoing support.

### Data collection

- *Fidelity assessment*: Adherence was evaluated via weekly structured observations using standardized checklists developed specifically for this study through a three-stage iterative process. First, operational definitions of each digital intervention’s core components were extracted from WHO-DIHS guidelines and local standard operating procedures. Second, observable behavioral indicators for each component were identified through consultations with technical experts and frontline health workers. Third, items were piloted and refined through two rounds of field testing. The final checklists comprised intervention-specific items assessing both process adherence (whether prescribed steps were followed) and quality indicators (completeness and accuracy of execution). For example, the Drug Stock Notification checklist included seven items, including inventory update frequency, data entry accuracy, alert threshold compliance and timely response to automated notifications. Each item was scored dichotomously (completed as specified/not completed), allowing calculation of percentage adherence scores. Trained research assistants achieved inter-rater reliability kappa coefficients exceeding 0.85 through standardized training and periodic reliability checks (Hallgren, 2012). These observations were triangulated with back-end analytics from digital platforms. Dose was measured through coverage (proportion of intended recipients reached), frequency (number of contacts) and duration (length of sessions), with data extracted from facility registers and platform analytics.
- *Implementation outcomes assessment*: Outcomes were assessed using validated instruments with strong psychometric properties. The Acceptability of Intervention Measure (AIM), Intervention Appropriateness Measure (IAM) and Feasibility of Intervention Measure (FIM) are parallel four-item instruments measured on a five-point Likert scale (Weiner et al., 2017). Organizational readiness was evaluated using the 12-item Organizational Readiness for Implementing Change (ORIC) scale, which assesses change commitment and change efficacy (Shea et al., 2014). These instruments were administered at baseline and post-implementation.
- *Qualitative data collection*: To explore implementation experiences and the perceived advantages and disadvantages of the digital initiatives (Chen and Gombay, 2024), semi-structured interviews were conducted with purposively sampled health workers (*n* = 30) and clients (*n* = 20). Interview guides were developed using the Theoretical Domains Framework to probe for barriers and facilitators related to knowledge, skills and environmental context (Cane et al., 2012). Interviews were conducted in participants’ preferred language (English or Yoruba), audio-recorded, transcribed verbatim and continued until thematic saturation was achieved (Guest et al., 2020).

### Data analysis plan

*Quantitative analysis*: Fidelity scores were calculated as percentages for adherence and dose measures, with a predetermined threshold of ≥80% considered high fidelity. Data from the AIM, IAM, FIM and ORIC scales were analyzed using SPSS version 25.0. Descriptive statistics were used to characterize participant demographics and outcome distributions, while bivariate analyses (Pearson correlations and chi-square tests) were used to examine associations between fidelity and implementation outcomes.

*Qualitative analysis*: Interview transcripts underwent thematic analysis following Braun and Clarke’s (2006) six-phase approach. Two researchers independently coded all transcripts using NVivo 12 software. An initial codebook was developed deductively based on the study’s theoretical frameworks and then expanded inductively to capture emergent themes, particularly those related to stakeholder perceptions and concerns, which can influence resistance to mHealth solutions (Fox et al., 2020). Inter-rater reliability was assessed, with disagreements resolved through consensus.

*Mixed-methods integration*: A convergent triangulation approach was used. Quantitative fidelity and implementation outcome data were systematically integrated with qualitative findings using joint displays and narrative synthesis to develop a comprehensive understanding of the implementation processes and to use the qualitative data to explain patterns observed in the quantitative results (Guetterman et al., 2015).

### Ethical considerations

Ethical approvals were obtained from the Research and Ethics Committee of Lagos State University Teaching Hospital (LREC/06/10/1294) and the WHO Ethics Review Committee (Protocol ID ERC.0003300; Version 2.0). The study adhered to the ethical principles of the Declaration of Helsinki. Written informed consent was obtained from all participants before data collection. Data confidentiality was maintained through the use of unique identifiers and secure, encrypted data storage protocols.

## Results

### Participant characteristics and facility context

A total of 55 health workers across the five PHCs participated in the study. This cohort was predominantly female (60.0%), with a mean age of 38.7 years (standard deviation = 7.2) and an average of 9.4 years of professional experience. The sample comprised PHC doctors (18.2%), nurses and midwives (45.5%), community health officers (18.2%) and pharmacy technicians (18.2%). A notable baseline characteristic was the relatively low self-reported experience with digital health tools for clinical purposes; only 22.0% of health workers indicated prior use beyond basic communication applications.

The 150 recruited client participants presented with a range of common mental disorders, primarily depression (45.3%), anxiety disorders (32.0%) and psychosis (18.7%). Among this group, 67.3% reported owning a smartphone. However, only 34.0% had ever used a dedicated health application, highlighting a potential gap in digital health literacy among the service user population.

The five participating PHCs varied significantly in their implementation context. Two facilities were located in high-density urban areas, benefiting from relatively stable electricity (~18–20 h per day) and reliable 4G network connectivity. In contrast, the remaining three were in peri-urban settings, characterized by greater infrastructure challenges, including less reliable power (~12–16 h per day) and inconsistent network connectivity. Facility logs documented that these infrastructure deficits directly impacted the implementation of synchronous interventions, with frequent service disruptions. This urban/peri-urban divide in infrastructure served as a key moderating factor in the implementation outcomes, as detailed in Table 1.

**Table 1.** Participant and facility characteristics

Characteristic	Category	Value
Health workers (*n* = 55)		
Mean age (SD), years		38.7 (6.2)
Gender	Female	33 (60.0%)
	Male	22 (40.0%)
Professional cadre	Nurse/midwife	25 (46.05%)
	Community Health Officer	10 (18.0%)
	PHC doctor	10 (18.0%)
	Pharmacy technician	10 (18.0%)
Mean years of experience (SD)		9.4 (4.5)
Prior digital health experience	Yes	12 (21.8%)
Clients (*n* = 150)		
Primary diagnosis	Depression	68 (45.3%)
	Anxiety disorder	48 (32.0%)
	Psychosis	28 (18.7%)
	Other	6 (4.0%)
Mean age (SD), years		41.2 (11.5)
Gender	Female	95 (63.3%)
Smartphone ownership	Yes	101 (67.3%)
PHC facilities (*n* = 5)		
Location	Urban	2 (40.0%)
	Peri-urban	3 (60.0%)
Average daily electricity (SD), hours		15.6 (2.5)
Predominant network coverage	4G	2 (40.0%)
	3G/intermittent	3 (60.0%)

*Note:* Percentages are rounded to one decimal place. SD, standard deviation.

### Quantitative findings: A bifurcation in implementation success

The quantitative data revealed a striking divergence in the implementation of the five digital health recommendations. A clear pattern emerged wherein administrative tools that streamlined existing processes were implemented successfully, while clinical tools that sought to modify direct patient interaction faced significant challenges. This pattern was consistent across both fidelity metrics and the implementation outcome scores.

#### Implementation fidelity

**Figure 1.**
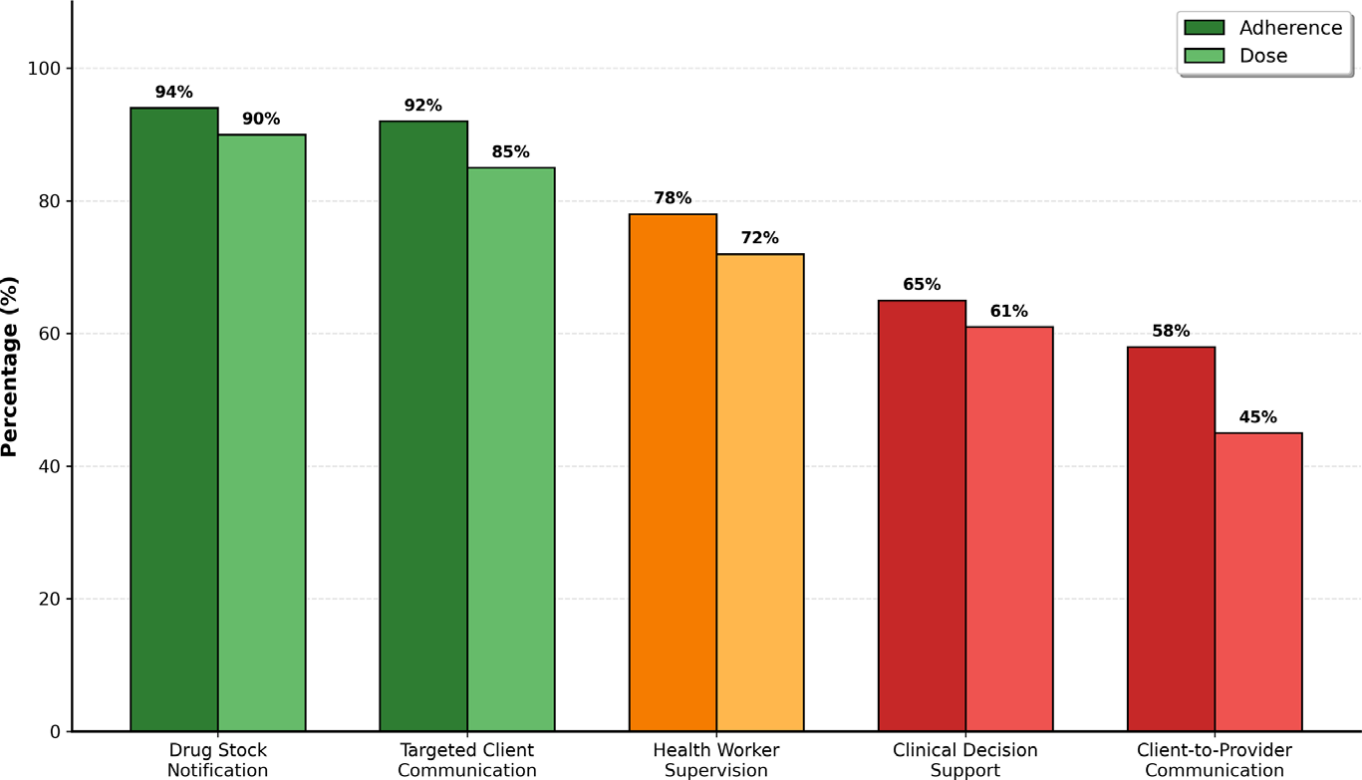
Implementation fidelity of digital interventions: Mean adherence and dose percentage across five PHC facilities.

Using a predetermined benchmark of ≥80% for high fidelity, the analysis of adherence (content delivered as intended) and dose (coverage and frequency) revealed the following:

- *High-fidelity interventions*: The two administrative interventions demonstrated high fidelity. *Drug Stock Notification* achieved the highest mean adherence score of 94% across the five facilities, with 89.5% of required reports completed (dose). *Targeted Client Communication* also showed high fidelity, with an adherence score of 92% for sending scheduled appointments and medication reminders, successfully reaching 85% of eligible clients.
- *Moderate-fidelity intervention*: *Health Workers’ Supervision* via digital platforms achieved a moderate mean adherence score of 78.4%. While most scheduled sessions occurred, observations and logs revealed that ~21.6% were either rescheduled, shortened or canceled due to technical difficulties, particularly in peri-urban sites.
- *Low-fidelity interventions*: The two interventions involving direct clinical interaction were implemented with low fidelity. *Clinical Decision Support*, which involved using the e-mhGAP-IG during consultations, had a mean adherence score of 65.1%. Direct observations indicated that health workers consulted the tool in approximately two-thirds of eligible patient encounters. *Client-to-Provider Communication* via the dedicated helpline had the lowest fidelity, with a mean adherence score of 58.3%, reflecting that health workers responded to incoming client communications consistent with protocol in just over half of instances. Figure 1 provides a visual summary of these divergent fidelity rates.

Further analysis revealed a strong, statistically significant negative correlation between the perceived complexity of an intervention and its adherence rate (*r* = −0.73, *p* < 0.001), suggesting that as interventions required more intricate steps or behavioral changes, they were implemented less faithfully.

#### Implementation outcomes

Stakeholder perceptions of the interventions, as measured by the AIM, IAM and FIM scales, largely mirrored the fidelity findings. The *ORIC scale* indicated a high level of overall readiness before implementation, with a mean score of 55.6 out of 60, suggesting a shared organizational commitment to the project’s goals at baseline. However, this high readiness did not translate into positive outcomes for all interventions. Administrative tools received significantly higher scores for acceptability, appropriateness and feasibility than the clinical tools. For example, the *Drug Stock Notification* tool was rated by pharmacy technicians as highly acceptable (80.5%), appropriate (90.0%) and feasible (86.1%). In contrast, the *Client-to-Provider Communication* helpline received the lowest scores from health workers, who rated it as having low acceptability (72.0%) and appropriateness (66.2%). Tellingly, clients who used the service rated its acceptability much higher (86.0%), highlighting a significant perceptual gap between providers and service users regarding the intervention’s value and safety, as detailed in Table 2.

**Table 2.** Stakeholder perceptions of implementation outcomes for digital health interventions

Digital intervention	Stakeholder group	Acceptability (AIM)	Appropriateness (IAM)	Feasibility (FIM)
		Mean score (%)	Mean score (%)	Mean score (%)
Drug Stock Notification	Pharmacy technicians	16.1 (80.5%)	18.0 (90.0%)	17.2 (86.1%)
	Health managers	16.8 (84.1%)	17.2 (86.2%)	18.0 (90.0%)
Targeted Client Communication	Clients	18.2 (91.0%)	17.3 (86.6%)	17.3 (86.5%)
Health Workers’ Supervision	Health workers	16.8 (84.0%)	17.2 (86.1%)	17.5 (87.6%)
	Health managers	16.2 (81.0%)	16.8 (84.0%)	16.4 (81.7%)
Clinical Decision Support	Health workers	**15.8 (79.0%)**	16.3 (81.5%)	17.3 (86.5%)
Client-to-Provider Communication	Health workers	**14.4 (72.0%)**	**13.2 (66.2%)**	**14.6 (73.0%)**
	Clients	17.2 (86.0%)	18.2 (91.2%)	16.7 (83.5%)

*Note*: Scores are out of a maximum of 20. Percentages are calculated from the maximum possible score. Scores below 80% are bolded to indicate lower performance.

*Technical requirements and phone type utilization.* While the targeted client communication intervention was initially designed with smartphone functionality in mind, post-implementation analysis revealed that high fidelity was achieved through a multimodal delivery approach accommodating varying phone capabilities. Among the 150 client participants, 101 (67.3%) reported owning smartphones, while 49 (32.7%) possessed basic feature phones with SMS capability. The intervention was delivered through WhatsApp for smartphone owners and standard SMS for basic phone users, maintaining identical message scheduling, content and frequency across both platforms. Platform analytics demonstrated equivalent effectiveness across phone types for core intervention components. Message delivery rates were statistically equivalent between platforms (SMS: 96.2% vs. WhatsApp: 97.1%, *χ*^2^ = 0.23, *p* = 0.63), indicating reliable message receipt regardless of phone sophistication. Client-reported appointment attendance following reminder messages showed no significant difference by phone type (basic phone: 83.7% [41/49] vs. smartphone: 86.4% [87/101], *χ*^2^ = 0.28, *p* = 0.48). Similarly, self-reported medication adherence following reminder prompts demonstrated comparable rates (basic phone: 81.6% [40/49] vs. smartphone: 84.2% [85/101], *χ*^2^ = 0.21, *p* = 0.52). Qualitative interviews with clients (*n* = 20) revealed consistent perceptions of helpfulness and acceptability across phone types. Smartphone users (*n* = 14) noted appreciation for enhanced features, including message delivery confirmation and the ability to save psychoeducational images. Basic phone users (*n* = 6) emphasized the simplicity and reliability of SMS delivery. No clients expressed dissatisfaction with their assigned delivery modality.

### Integrated analysis: Explaining the divergence with qualitative data

The triangulation of quantitative data with qualitative findings from 50 stakeholder interviews provides a clear explanatory model for the observed variations in fidelity and acceptability. The primary qualitative themes that furnish this explanation are summarized in Table 3. The qualitative data revealed that the success or failure of an intervention was not primarily determined by its technological sophistication but by its interaction with existing human systems of work and professional identity.

**Table 3.** Key qualitative themes regarding barriers and facilitators to implementation

Theme	Description	Illustrative quote
1. Workflow integration vs. disruption	The degree to which a digital tool simplified and automated an existing task (facilitator) versus adding new steps or altering established clinical routines (barrier).	*“Before, I spent half of Monday counting drugs and writing reports. Now, I just update the tablet as I dispense, and the report is done. It removed work, it didn’t add it.”* – Pharmacy technician
2. Professional role and liability concerns	Anxiety and uncertainty among health workers regarding how digital tools impact their professional responsibilities, scope of practice and clinical accountability, particularly in high-risk situations.	*“What if a patient sends a message saying they want to die, and my network is down? The responsibility is too much. I cannot be held liable for what happens over WhatsApp.”* – PHC doctor
3. Infrastructure reliability	The foundational dependence on consistent electricity and network connectivity. While a universal factor, its impact varied based on whether an intervention required synchronous (real-time) or asynchronous operation.	*“The digital supervision is a good idea, but half the time the video freezes or the call drops. We spend more time saying ‘Can you hear me?’ than discussing the patient.”* – Community Health Officer
4. Digital literacy and technical support	The confidence and skill of health workers in using the new technology, and the perceived availability and responsiveness of technical support to troubleshoot problems, which mediated user frustration.	*“At first, it was hard, but the support person was very patient. After he showed me twice, I understood it. Knowing I could call him made me less afraid to try.”* – Nurse

#### High-fidelity interventions: Explained by seamless workflow integration

The success of the *Drug Stock Notification* and *Targeted Client Communication* systems was overwhelmingly attributed to their seamless integration into, and simplification of, existing workflows. Health workers did not perceive these tools as new, burdensome tasks but as more efficient methods for completing routine responsibilities that directly addressed known “pain points.”

*Before, I spent half of Monday counting drugs and writing reports. Now, I just update the tablet as I dispense, and the report is done. It removed work, it didn’t add it*. – Pharmacy Technician

Similarly, the automated nature of the client messaging system was seen as a major facilitator, as it enhanced patient communication without adding to the clinical workload. This sentiment of the intervention being a “solution to an existing problem” directly explains their high fidelity and acceptability scores.

#### Low-fidelity interventions: Explained by workflow disruption and professional risk

Conversely, the two low-fidelity interventions were consistently described as disruptive to established clinical practices and generative of new professional anxieties.

- *Clinical Decision Support:* The low adherence (65.1%) to the e-mhGAP-IG was directly explained by the qualitative theme of workflow disruption. Many health workers, particularly more experienced nurses, felt that consulting a device during a patient encounter was awkward and detrimental to building rapport, perceiving it as a barrier to the therapeutic alliance.
*Looking at my phone makes me feel like I don’t know my job. The patient will think I am not paying attention. I prefer to have the chart on the wall where I can glance at it. The phone feels… rude*. – Nurse
- *Client-to-Provider Communication:* The very low fidelity (58.3%) of this intervention was powerfully explained by the dominant qualitative theme of professional role and liability concerns. Over 90% of interviewed health workers expressed significant anxiety about managing clinical emergencies, particularly suicide risk, via a digital platform without the benefit of a face-to-face assessment. This fear was compounded by the lack of a clear policy for reimbursement or clinical liability.
*What if a patient sends a message saying they want to die, and my network is down? The responsibility is too much. For an emergency, they must come to the clinic. I cannot be held liable for what happens over WhatsApp*. – PHC Doctor

This profound sense of perceived risk, combined with concerns about the blurring of work–life boundaries, created a powerful disincentive for engagement that high organizational readiness at the outset could not overcome. This integrated analysis demonstrates a clear pattern: digital health interventions that were perceived as simple, administratively efficient and aligned with existing professional roles were implemented with high fidelity. In contrast, interventions that were perceived as clinically complex, disruptive to the provider–patient dynamic, and that introduced new professional risks or uncertainties were implemented with low fidelity.

## Discussion

**Figure 2.**
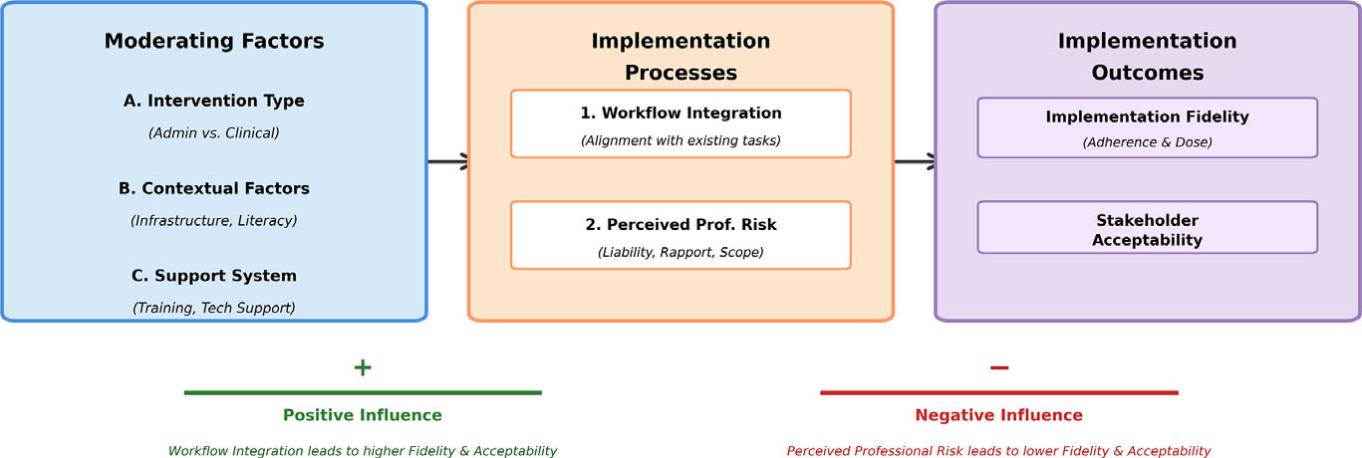
Conceptual model of factors influencing digital health implementation.

This process evaluation of a pilot digital health implementation in Nigerian primary care provides a nuanced and critical understanding of the factors that mediate the translation of evidence-based guidelines into real-world practice (Figure 2). Our integrated mixed-methods analysis revealed a striking divergence in the implementation success of different digital health recommendations, a pattern that was not predicted by the high organizational readiness observed at baseline. The findings suggest that the nature of the intervention itself, specifically its relationship with existing clinical workflows and professional roles, may be a more potent determinant of implementation fidelity than the preexisting motivation of health system actors.

### Interpretation of key findings and comparison with existing literature

The central finding of this study is the clear bifurcation in fidelity between administrative and clinical digital tools. Interventions designed to enhance efficiency in routine, nonclinical tasks, such as drug stock notification and automated client messaging, were implemented with high fidelity and were well-received by stakeholders. This success strongly resonates with the broader literature on the diffusion of innovations, which posits that the perceived complexity and compatibility of an innovation are critical predictors of its adoption (Rogers, 2003). It also aligns with local evidence; studies conducted within Nigeria have consistently shown that mobile phone interventions, such as call or text message reminders, are effective at improving treatment engagement and adherence (Adewuya et al., 2019b; Elugbadebo et al., 2023). The success of our administrative tools appears to stem from their ability to directly address existing pain points without requiring significant behavioral change. This supports a core tenet of Normalization Process Theory, which posits that for a new practice to become embedded, participants must be able to see its value and integrate it coherently into their existing work (May and Finch, 2009).

In stark contrast, the low-fidelity clinical interventions appear to represent a classic case of implementation failure driven by a lack of compatibility and perceived complexity. The resistance to the clinical decision support tool, for instance, speaks to a well-documented challenge in health IT implementation: the disruption of the fluid dynamics of the clinical encounter (Greenhalgh et al., 2004). Health workers’ concerns that consulting a device felt “rude” and undermined their professional rapport with clients are consistent with literature highlighting the potential for technology to interfere with the therapeutic alliance (Montague et al., 2013). While the protocol for a feasibility trial of the e-mhGAP-IG app in Nigeria noted the potential to enhance quality of care, it also underscored the need to assess its feasibility and acceptability in practice (Taylor Salisbury et al., 2021); our findings provide critical evidence on these points, suggesting significant real-world barriers to its adoption.

Perhaps the most critical finding is the powerful role that *professional role and liability concerns* played in the failure of the client-to-provider communication intervention. The profound anxiety expressed by health workers regarding the management of clinical emergencies via digital platforms is a significant barrier that transcends simple technical training. This aligns with global evidence on telehealth adoption, where medical liability and patient safety in remote interactions remain primary concerns for clinicians (Scott Kruse et al., 2018). Our study contributes a vital perspective from an LMIC setting, demonstrating that without clear systemic support, task-shifting digital mental health support introduces a level of perceived risk that may be untenable for frontline workers. This finding is powerfully contextualized by recent qualitative work in Nigeria, which identified a lack of supportive policies, skepticism and concerns about sustainability as major disadvantages hindering digital mental health initiatives (Chen and Gombay, 2024). The divergence in perception between clients, who found the service highly acceptable, and providers, who did not, is also a noteworthy theme. It suggests high patient interest in mHealth, which has been widely reported across diverse Nigerian populations, including perinatal adolescents (Kola et al., 2021; 2025), older adults with mental illness (Elugbadebo et al., 2023), young urban populations (Akiogbe et al., 2024) and university students (Ogundipe et al., 2025), with similar patterns observed across sub-Saharan Africa (Barkley et al., 2025). This, however, does not automatically translate into a feasible or safe service from the provider’s perspective without an adequate policy and support infrastructure.

Finally, while *infrastructure and digital literacy* are widely acknowledged as foundational barriers in sub-Saharan Africa (Karamagi et al., 2022), our study provides a more granular understanding of their role. We found that infrastructure reliability did not uniformly affect all interventions. Asynchronous tools were resilient to intermittent connectivity, whereas synchronous tools like digital supervision were directly hampered by it, particularly in peri-urban facilities. This demonstrates that a blanket assessment of infrastructure is insufficient; instead, a “fit-for-purpose” analysis is needed, matching the technical requirements of a specific digital tool to the realities of the local context.

### Theoretical contributions

This study makes several contributions to implementation science theory. First, by applying the Carroll et al. (2007) fidelity framework to a multicomponent digital health intervention in an LMIC, we provide a robust empirical example of its utility in this context. More importantly, our findings suggest a potential refinement of the framework for digital health. While the framework identifies “intervention complexity” as a key moderator, our results indicate that this concept may need to be disaggregated. We found that *technical complexity* (e.g., learning to use an app) was a manageable barrier. In contrast, *workflow complexity* (the degree to which an intervention disrupts established clinical practice) and *professional complexity* (the extent to which it introduces new risks and role ambiguities) were far more powerful and less tractable determinants of fidelity. We propose that for digital health interventions, these dimensions of complexity should be assessed independently, as they appear to have differential impacts on implementation success.

Second, our findings highlight the limitations of relying solely on upstream measures like organizational readiness as a predictor of success. The high ORIC scores at baseline demonstrated a genuine willingness to change, yet this did not translate to successful implementation for the more challenging clinical interventions. This suggests that while readiness may be a necessary condition, it is not sufficient. The characteristics of the intervention and its interaction with the professional context at the point of care can override even strong organizational motivation. This reinforces the socio-technical perspective that implementation is an emergent process shaped by the dynamic interplay between the technology, the user and the organizational system, rather than a linear deployment of a tool into a willing environment (Sittig and Singh, 2010).

### Limitations

This study has several limitations that should be acknowledged. First, the 3-month pilot period, while sufficient for assessing initial fidelity, does not allow for an evaluation of long-term sustainability. Second, the study was conducted in Lagos State, which has a more developed infrastructure than many other parts of Nigeria; the findings may not be generalizable to more rural or resource-poor settings. Third, as this was a process evaluation without a control group, we cannot make causal claims about the effectiveness of the interventions on clinical or service delivery outcomes. Finally, the presence of research staff may have introduced a Hawthorne effect, potentially inflating fidelity scores, although we sought to mitigate this by triangulating data with nonobtrusive platform analytics.

### Implications for practice and policy

Despite these limitations, our findings offer several clear and actionable implications.

- *For implementers*: A phased and strategic approach is recommended. Begin with “low-hanging fruit”; the administrative interventions that solve clear problems with minimal disruption. For more complex clinical tools, a user-centered, codesign process that actively involves frontline health workers is essential to ensure workflow compatibility and address professional concerns from the outset. This approach is supported by other Nigerian studies, which have successfully used theory-driven, stakeholder-engaged methods to design mHealth interventions (Kola et al., 2022; 2025).
- *For policymakers*: Successful digital health implementation requires a supportive policy ecosystem. Our findings point to an urgent need for clear national or state-level guidelines on tele-consultation, addressing critical issues of clinical liability, data privacy and the scope of practice for primary care workers. As recent reviews have noted, policies to operationalize digital healthcare services are critical for addressing unmet mental health needs in Nigeria (Onu and Onyeka, 2024). Without this “policy infrastructure,” frontline workers will continue to bear a level of risk that will understandably suppress adoption (Chen and Gombay, 2024). Furthermore, investments in foundational public infrastructure, particularly reliable power and internet connectivity, must be seen as integral components of any national digital health strategy.

*The imperative of technology-agnostic design for digital health equity*: The finding that high implementation fidelity (92% for targeted communication, 85% for dose coverage) was achieved despite only 67.3% smartphone ownership challenges prevailing assumptions about necessary technological thresholds for digital health success. Our phone type utilization analysis revealed no significant differences in message delivery rates, appointment attendance or medication adherence between basic phone and smartphone users. This finding has profound implications for how we conceptualize and design digital health interventions in resource-limited settings. These results demonstrate that the core function of targeted client communication depends primarily on reliable text message delivery, which basic feature phones adequately provide. While smartphones enable enhanced features, such as multimedia content, read receipts and interactive elements, these represent value-added functionality rather than essential mechanisms of action. The initial specification of smartphone requirements in WHO-DIHS recommendations may, therefore, be modifiable based on local context without compromising implementation fidelity or clinical effectiveness. Implementation scientists and policymakers should distinguish between “optimal” and “sufficient” technology requirements when designing and evaluating digital health interventions. This distinction carries substantial implications for digital health equity in LMICs. Viewing insufficient smartphone penetration as an implementation barrier requiring resolution before intervention deployment risks delaying potentially beneficial services and inadvertently widening health inequities by excluding economically disadvantaged groups with limited access to newer technologies. For resource-limited settings like Nigeria, where smartphone penetration remains incomplete, designing digital health interventions with backward compatibility to basic SMS functionality may substantially extend population reach without sacrificing effectiveness. Our findings support an alternative “technology-agnostic” design philosophy, wherein interventions are engineered to deliver core functions through the lowest common technological denominator while offering enhanced experiences for users with more sophisticated devices. This approach aligns with broader digital health literature suggesting that intervention effectiveness depends less on technological sophistication than on alignment with user needs, workflows and capabilities (Onu and Onyeka, 2024). Recent scoping reviews of digital psychiatry in Nigeria have emphasized the importance of contextually appropriate technology selection that considers existing infrastructure and user capabilities. Similarly, studies examining digital health integration across multiple African countries have demonstrated that successful implementation requires matching technological complexity to local capacity (Barkley et al., 2025). From a health systems perspective, backward compatibility to basic SMS functionality serves as a form of technological insurance, ensuring that interventions remain functional even when infrastructure challenges (power outages, network instability and device obsolescence) temporarily degrade access to smartphone-dependent features. This resilience may be particularly valuable in settings characterized by infrastructural unpredictability, where maintaining service continuity is paramount. Our finding that clients expressed satisfaction with their assigned modality regardless of phone type suggests that users prioritize functional utility over technological sophistication when health outcomes are at stake.

## Conclusion

This process evaluation provides a granular, theory-driven account of the complexities of implementing digital health guidelines in a real-world LMIC setting. It serves as a crucial reminder that technology is not a panacea. The success of digital health interventions is not predetermined by the sophistication of the tool or the motivation of the workforce, but by the degree to which they are thoughtfully integrated into the human systems of healthcare. By distinguishing between interventions that simplify work and those that add complexity and risk, this study offers an evidence-based roadmap for implementers and policymakers seeking to harness the power of digital technology to strengthen mental health systems and close the treatment gap in Nigeria and beyond.

## Data Availability

The data supporting the findings of this study are available upon reasonable request from the corresponding author. Due to ethical and privacy considerations, de-identified datasets can be shared with qualified researchers, subject to approval from the Lagos State Ministry of Health and the ethics committee.
