# Supplementary material for: Fidelity and feasibility of a pilot implementation of adapted WHO digital interventions to scale up mental health in Nigerian primary care: A mixed-methods process evaluation
Source: Glob Ment Health (Camb). 2026 Jan 22;13:e65. doi: 10.1017/gmh.2026.10131 (PMC13112290; doi:10.1017/gmh.2026.10131)
Supplement: Peer review history

# Editor report: Recommendation: Fidelity and feasibility of a pilot implementation of adapted WHO digital interventions to scale up mental health in Nigerian primary care: A mixed-methods process evaluation — R1/PR2

**DOI:** 10.1017/gmh.2026.10131.pr2

**Affiliations:** Centre for Rural Health, University of KwaZulu-Natal, South Africa

No accompanying comment.

# Editor report: Decision: Fidelity and feasibility of a pilot implementation of adapted WHO digital interventions to scale up mental health in Nigerian primary care: A mixed-methods process evaluation — R1/PR3

**DOI:** 10.1017/gmh.2026.10131.pr3

**Affiliations:** London School of Hygiene & Tropical Medicine, United Kingdom of Great Britain and Northern Ireland

No accompanying comment.
